# Comparison of 27-gauge beveled-tip and 25-gauge flat-tip microincision vitrectomy surgery in the treatment of proliferative diabetic retinopathy: a randomized controlled trial

**DOI:** 10.1186/s12886-023-03251-2

**Published:** 2023-12-12

**Authors:** Jingjie Liu, Boshi Liu, Juping Liu, Dejia Wen, Manqiao Wang, Yan Shao, Xiaorong Li

**Affiliations:** 1 Tianjin Key Laboratory of Retinal Functions and Diseases, Tianjin Branch of the National Clinical Research Center for Ocular Disease, Eye Institute, School of Optometry, Tianjin Medical University Eye Hospital, 251 Fukang Road, Tianjin, China; 2 Tianjin Medical University Eye Hospital, 251 Fukang Road, 300384 Tianjin, China

**Keywords:** Vitrectomy, Diabetic retinopathy, Vitreoretinal Surgery

## Abstract

**Purpose:**

To compare the effectiveness and safety of a 27-gauge (27G) beveled-tip microincision vitrectomy surgery (MIVS) with a 25-gauge (25G) flat-tip MIVS for the treatment of proliferative diabetic retinopathy (PDR).

**Methods:**

A prospective, single-masked, randomized, controlled clinical trial included 52 eyes (52 patients) with PDR requiring proliferative membrane removal. They were randomly assigned in a 1:1 ratio to undergo the 27G beveled-tip and or 25G flat-tip MIVS (the 27G group and the 25G group, respectively). During surgery, the productivity of cutting the membrane, the number of vitrectomy probe (VP) exchanges to microforceps, total operation time, vitrectomy time and intraoperative complications were measured. Best-corrected visual acuity (BCVA), intraocular pressure (IOP) and postoperative complications were also assessed to month 6.

**Results:**

Forty-seven eyes (47 patients) completed the follow-up, including 25 in the 27G group and 22 in the 25G group. During surgery in the 27G group, cutting the membrane was more efficient (*P* = 0.001), and the number of VP exchanges to microforceps was lower (*P* = 0.026). The occurrences of intraoperative hemorrhages and electrocoagulation also decreased significantly (*P* = 0.004 and *P* = 0.022). There were no statistical differences in the total operation time or vitrectomy time between the two groups (*P* = 0.275 and *P* = 0.372), but the former was slightly lower in the 27G group. Additionally, the 27G group required fewer wound sutures (*P* = 0.044). All the follow-up results revealed no significant difference between the two groups.

**Conclusions:**

Compared with the 25G flat-tip MIVS, the 27G beveled-tip MIVS could be more efficient in removing the proliferative membrane while reducing the occurrence of intraoperative hemorrhages and electrocoagulation using appropriate surgical techniques and instrument parameters. Its vitreous removal performance was not inferior to that of the 25G MIVS and might offer potential advantages in total operation time. In terms of patient outcomes, advanced MIVS demonstrates equal effectiveness and safety to 25G flat-tip MIVS.

**Trial registration:**

The clinical trial has been registered at Clinicaltrials.gov (NCT0544694) on 07/07/2022. And all patients in the article were enrolled after registration.

## Introduction

Pars plana vitrectomy (PPV) is a major surgical method for the treatment of vitreoretinal diseases that was originally introduced in 1971 with a 17-gauge system [1]. In the past 20 years, vitrectomy has gradually trended towards the use of smaller gauges and greater functionality [2]. This provides excellent advantages for surgical procedures and operative effects, especially in some complex cases, such as proliferative diabetic retinopathy (PDR) [3]. During vitrectomy for PDR, the most critical step is the removal of the proliferative membrane. The tight adhesion between the membrane and retina often makes it difficult for surgeons to begin the procedure. At the same time, traction to the retina during the operation easily causes hemorrhage and retinal breakage [4]. The current mainstream surgical method, microincision vitrectomy surgery (MIVS), can more easily enter the space between the membrane and retina with its smaller gauge [5]. However, it is more suitable for lesions near the retinal periphery, where the probe port can be closer to the issue. Substantial damage to the retina may still be caused when dealing with the membrane at the posterior pole. In addition, some scholars believe that a smaller gauge means less vitrectomy efficiency, which might affect the process of surgery [6]. In 2018, Dr. Chow reported a clinical observational study involving a novel 27G beveled-tip MIVS whose beveled-tip vitrectomy probe (VP) could serve as both a cutter and a delamination tool [7]. Combined with a port closer to the top, it can present with great utility for removing the membrane and reducing the steps of switching instruments during surgery. At the same time, the higher cutting rate may favorably affect deficiencies such as a low flow rate [8]. These seem to be potentially beneficial to vitrectomy, which requires delicate and complex surgical movements. However, as advanced VP has become available, there is still a serious lack of related research in the treatment of patients with PDR, and the efficiency of removing membranes during surgery has not yet been quantified. Currently, 25G flat-tip MIVs are still widely used in routine PDR operations worldwide. Thus, this study aimed to compare the effectiveness and safety of PDR treatments with the 27G beveled-tip MIVS and the conventional 25G flat-tip MIVS.

## Method

This was a prospective, single-masked, randomized, comparative study. Ethical approval was obtained from the Ethics Committee of Tianjin Medical University Eye Hospital (2021KY-27), and the study adhered to the tenets of the Declaration of Helsinki. The study was registered at Clinicaltrials.gov (NCT0544694).

### Participants

The study was conducted at Tianjin Medical University Eye Hospital. Participant inclusion criteria were as follows: (1) definite diagnosis of PDR requiring vitrectomy, (2) presence of a substantial area of proliferative membranes on the retina using fundus imaging or B-ultrasonography, (3) age ≥ 18 years, and (4) follow-up for at least 6 months after surgery. Participant exclusion criteria included (1) corneal lesions affecting the operative field, such as corneal opacity or scarring, (2) a prior history of vitreoretinal surgery, (3) external eye infections, (4) uncontrolled hypertension or hyperglycemia, (5) coagulation abnormalities or current use of anticoagulant drugs other than aspirin, (6) the inability to meet postoperative position requirements, (7) other ocular diseases that may damage visual acuity, and (8) irregular follow-up. Only one eye from each patient was included. Patients deemed eligible to participate were given detailed information about the study and successfully signed the written informed consent form. Then, the patients were randomized, and the assigned regimen was implemented.

### Procedures

The enrolled patients underwent a comprehensive ocular and general examination before surgery. Their detailed baseline information is shown in Table 1. The severity of proliferative membranes was graded according to previously published criteria: (1) Multiple-point adhesions, with or without one plaque-like adhesion; (2) Broad adhesions in less than three sites, located posterior to the equator; (3) Broad adhesions in more than three sites, located posterior to the equator, or extending beyond the equator within one quadrant; (4) Broad adhesions extending beyond the equator for more than one quadrant [9]. The patients were randomly assigned in a 1:1 ratio to group A (vitrectomy with 27G beveled-tip VP) and group B (vitrectomy with 25G flat-tip VP) by drawing lots. Allocation was performed before the operation, and all patients were masked to the assignment. The identified information was provided to the surgeons and the researchers before surgery but remained inaccessible to the patients.

All operations were performed by two experienced fundus surgeons according to the relevant standardized procedures. Group A was treated with a 27G, 10,000 cuts per minute (cpm) beveled-tip vitrectomy system (27G Advanced Ultravit, CONSTELLATION Vision System, Alcon Surgical, Irvine, CA, USA), and Group B was treated with a 25G, 7500 cpm flat-tip vitrectomy system (25G Ultravit, CONSTELLATION Vision System, Alcon Surgical, Irvine, CA, USA). Three days before vitrectomy, the surgeons evaluated the patients’ fundus condition. If there was a massive vitreous hemorrhage, or dense neovascularization on the proliferative membranes or retina, the patients would receive anti-vascular endothelial growth factor (anti-VEGF) treatment. Then, during the operation, the surgical microscope combined with the wide-field contact lens provided fundus observation, with matching 25G or 27G fibre-optic light providing endoillumination. The surgeon inserted three trocars through the sclera 3.5 to 4 mm posterior to the limbus. Core vitrectomy was performed at the maximum cutting rate, an intraoperative pressure (IOP) of 25 mmHg, and aspiration pressure of 650 mmHg. Then, the peripheral vitreous was removed with scleral depression applied by an assistant. The surgeon removed the proliferative membrane with VP and microforceps to release the traction on the retina. During this process, the VP was set to the maximum cutting rate, and the IOP and aspiration pressure were adjusted according to the surgeon’s need. Typically, the aspiration pressure was maintained within the range of 300–600 mmHg. In the case of retinal detachment, the patient also underwent fluid-air exchange, and an appropriate endotamponade substance was used. For patients who required intravitreous silicon oil injection, the 25G CONSTELLATION® Vision System VFC Syringe (Alcon Surgical, Irvine, CA, USA) and the 27G VFI cannulas (MedOne Surgical, Inc in Sarasota, FL, USA) were utilized in the two groups separately. Intraoperative cataract surgery, retinal laser photocoagulation, electrocoagulation and other treatments were performed in accordance with the conditions of the patient’s lens and fundus. At the end of surgery, the surgeon decided whether to suture the incision based on the presence of leakage. After surgery, the patients with retinal detachment were advised to maintain a strict facedown position for 1 week.

**Fig. 1:**
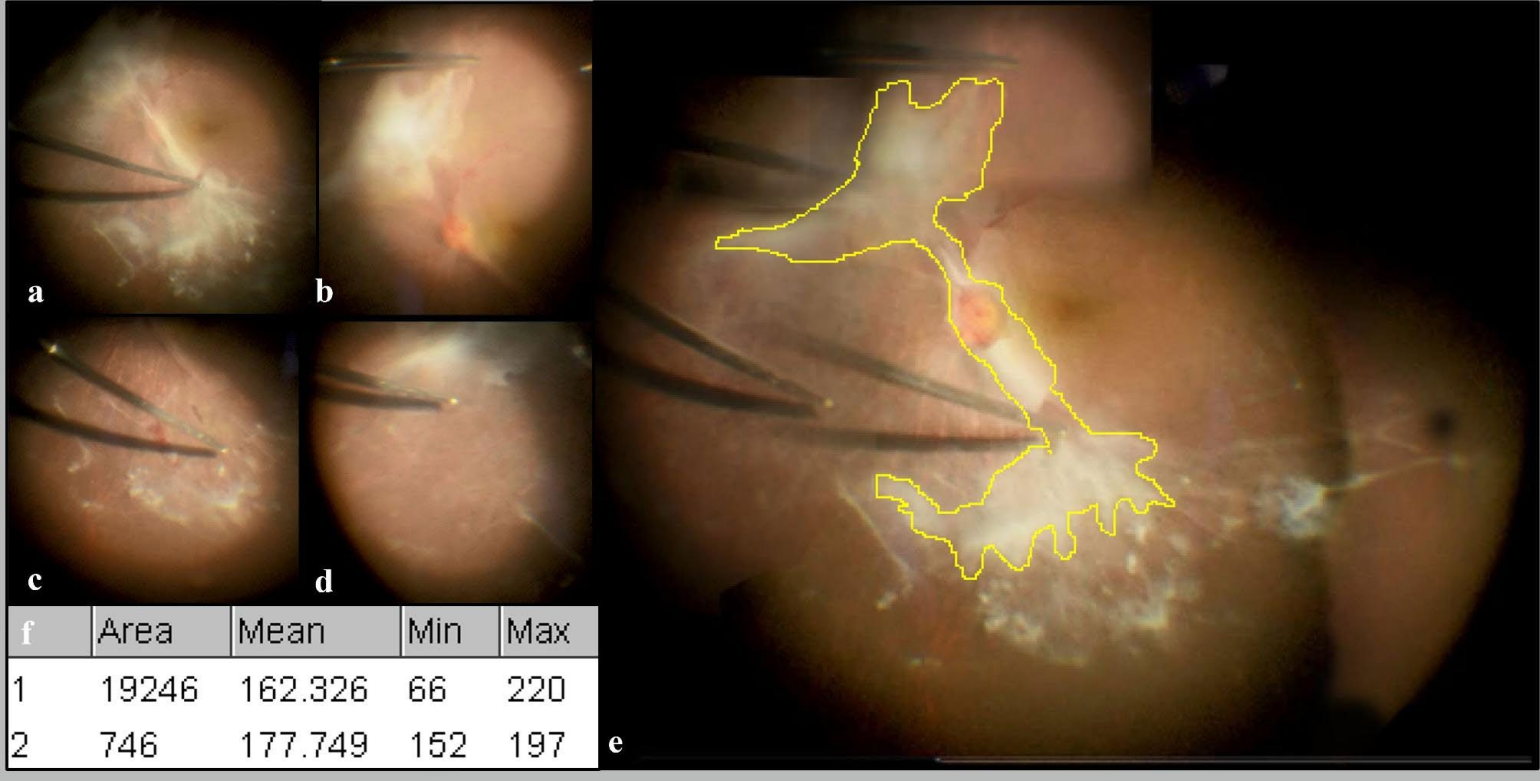
The retinal images captured during the surgery. (**a**)-(**d**) are the partial images in different fields under the microscope. (**e**) is the panoramic fundus image composed of multiple images by Photoshop. The part enclosed by the yellow line is the area of the proliferative membrane removed by VP in the operation. The proliferative membrane except that in the yellow line area was directly removed during vitrectomy; therefore, it was not included in the total area calculation. (**f**) is the measurement result showing the area of the membrane (1) and optical disc (2) quantified by ImageJ, expressed in pixels

The operative efficiency was mainly determined by the total operation time, core vitrectomy time and productivity of cutting the membrane. The total operation time was defined as the time interval from the insertion of the first trocar to the closure of all the incisions. Core vitrectomy time was the time to clear the core vitreous within the visible range provided by the HR Direct 1X Lens (Volk ,Mentor ,OH ,USA). The productivity of cutting the membrane was quantified, and the method is shown in Fig. 1. The partial retinal images in different fields under a microscope were first captured in the surgical video. They were then assembled into a panoramic fundus image by Adobe Photoshop. ImageJ was then used to quantify the area of the total proliferative membrane removed by VP and the area of optic disc. The ratio of the two was the relative area of the membrane with respect to the area of optic disc. Finally, by recording the cutting time for all the membranes from the video, the productivity was calculated and described in optic discs/min.

Patients were generally asked to follow up at 1 day, 1 week, 1 month, 3 months and 6 months after surgery. BCVA was tested using a standard visual acuity chart, and the measured decimal visual acuity was converted to (logarithm of the minimal angle of resolution) logMAR acuity. IOP was measured by a noncontact tonometer. In addition, patients underwent anterior segment and fundus examinations by slit lamp microscopy and indirect ophthalmoscopy for vitreous hemorrhage, retinal detachment and other postoperative complications. The surgeon recommended appropriate anti-VEGF or retinal photocoagulation treatment based on the presence of persistent macular edema, neovascularization or recurrent hemorrhage, if necessary.

### Outcomes

The primary outcome measure was the productivity of cutting the membrane in optic discs/min. Meanwhile, the secondary outcome measures included the number of VP exchanges to microforceps, the number of iatrogenic retinal breaks and bleeding during the surgery, and the postoperative BCVA and IOP of the patients in each period.

### Statistical analysis

The sample size was calculated based on the primary outcome measures with two-sided significance α = 0.05 and power = 0.8. Statistical analyses were performed by using SPSS (IBM SPSS Statistics 25). It was assumed that the productivity of cutting the membrane would be 3 optic discs/min in the 27G group and 2 optic discs/min in the 25G group, with a standard deviation of 1.2 disc/min in both groups, resulting in 45 eyes. Anticipating a 10% dropout rate, the final sample size was determined to be 25 participants in each group, for a total of 50 participants.

All statistical analyses were performed with SPSS (IBM SPSS 25.0). The data with a normal distribution confirmed by the Shapiro‒Wilk test were described as the mean ± standard deviation and underwent a two-tailed t test between the two groups. Otherwise, the median (interquartile range) was used to describe the data, and the Kruskal‒Wallis test was used for comparison. For unordered categorical variables, proportion (%) was utilized, and the chi-square test/Fisher’s exact test was suitable for comparison between groups. Statistical analyses of patients’ BCVA and IOP in different periods were performed using generalized estimating equations (GEEs). P values less than 0.05 were considered to indicate statistical significance.

## Results

**Fig. 2:**
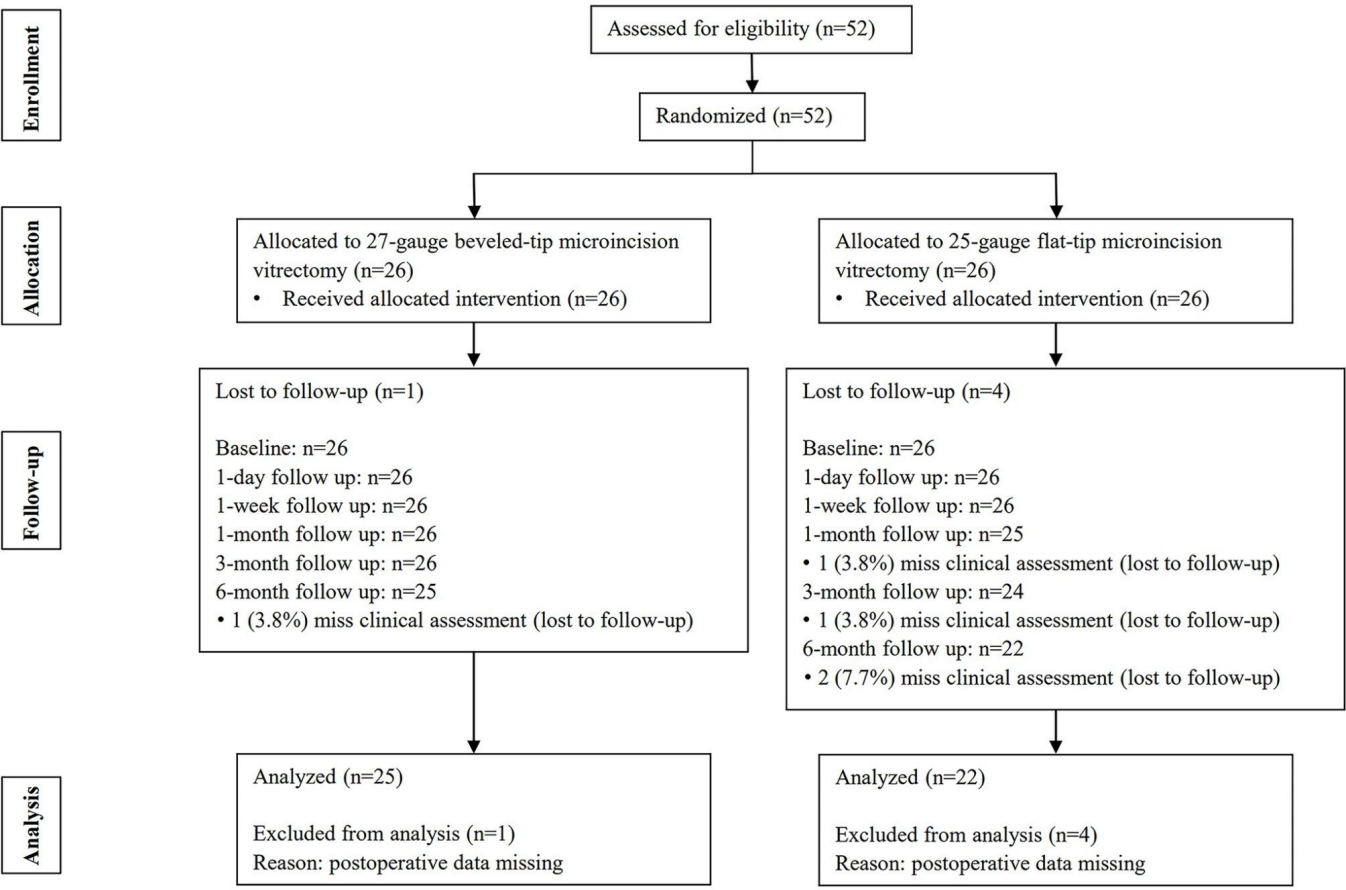
Flow diagram showing the randomization, follow-up and analysis of the intention-to-treat population

Fifty-two eyes (52 patients) were included, of which 26 eyes were randomly assigned to the 27G group and the others were assigned to the 25G group. Five patients (one in the 27G group and four in the 25G group) were lost to follow-up. The flow diagram is shown in Fig. 2. The descriptions and comparisons of the baseline characteristics between the two groups of patients are shown in Table 1. The differences were not statistically significant in age, sex, hemoglobin A1c (HbA1c), duration of diabetes mellitus (DM), grade of proliferative membrane, proliferative membrane involving macula, Number of retinal detachments, lens state, BCVA, IOP, anti-VEGF therapy, or panretinal photocoagulation (PRP) before surgery.

**Table 1:** Baseline Demographics and Clinical Data of the Two Groups

	27G beveled-tip (n = 25)	25G flat-tip (n = 22)	p value
Age (year)	52.2 ± 12.0	52.6 ± 8.7	0.906*
Male sex	12(48%)	12(54.5%)	0.503†
HbA1c (%)	7.9 ± 1.2	7.7 ± 1.5	0.544*
Duration of DM (year)	10 (10, 20)	10 (10, 20)	0.961ǂ
Grade			0.509†
I II III IV Macula involved	2 (8.0%) 10 (40.0%) 11 (44.0%) 2 (8.0%) 12 (54.5%)	4 (18.2%) 9 (40.9%) 6 (27.3%) 3 (13.6%) 10 (45.5%)	0.861†
Number of retinal detachments	19 (76.0%)	15 (68.2%)	0.550†
Lens status			1.000†
Phakic Pseudophakic Aphakic	23 (92.0%) 2 (8.0%) 0 (0%)	21 (95.5%) 1 (4.5%) 0 (0%)	
Preop BCVA (logMAR)	1.53 ± 0.49	1.68 ± 0.66	0.651*
Preop IOP (mmHg)	15.5 ± 3.2	16.4 ± 4.0	0.376*
Number of preop IVI Anti-VEGF treaments	17 (68.0%)	16 (72.7%)	0.724†
Number of preop PRP	2 (8.0%)	3 (13.6%)	0.880†

*Based on t-test; †based on chi-square test/Fisher’s exact test; ǂbased on Mann–Whitney U test.

27G, 27-gauge; 25G, 25-gauge; HbA1c, hemoglobin A1c; preop, preoperative; DM, diabetes mellitus; BCVA, preoperative best-corrected visual acuity; logMAR, logarithm of the minimal angle of resolution; IOP, preoperative intraocular pressure; IVI, intravitreal injection; Anti-VEGF, anti-vascular endothelial growth factor; PRP, panretinal photocoagulation.

### Surgical procedure

Intraoperative data acquired from surgical videos are shown in Table 2. The productivity of cutting the membrane in the 27G group was significantly greaterthan that in the 25G group (*P* = 0.001). Likewise, the number of VP exchanges to microforceps, one of the secondary outcome measures, was significantly lower in the 27G group (*P* = 0.026). Moreover, the 27G group had a slightly shorter operation time but a longer vitrectomy time than the 25G group, although neither of them were significantly different. In addition, the differences in other intraoperative measures, such as simultaneous cataract surgery and type of endotamponade substances, did not reach statistical significance, except for a lower percentage of patients who underwent suturing after surgery in the 27G group (*P* = 0.044).

**Table 2:** Surgical Procedure of the Two Groups

	27G beveled-tip (n = 25)	25G flat-tip (n = 22)	*p* value
Total time (min)	76.15 ± 21.38	83.19 ± 22.97	0.257*
Number of VP exchanges to microforceps (n)	0.54 ± 0.18	0.88 ± 0.16	0.026*
Productivity of cutting the membrane (optic discs/min)	2.45 ± 0.67	1.43 ± 0.34	0.001*
Core vitrectomy time (min)	2.22 (1.33, 2.55)	1.75 (1.48, 1.88)	0.372ǂ
Number undergoing simultaneous cataract surgery	15 (60.0%)	17 (77.3%)	0.119†
Endotamponade substance			0.257†
None Room air C3F8 Silicone oil	11 (44.0%) 1 (4.0%) 2 (8.0%) 11 (44.0%)	6 (27.3%) 0 (0%) 5 (22.7%) 11 (50.0%)	
Number requiring wound sutures	2 (8.0%)	8 (21.3%)	0.044*†*

*Based on t-test; †based on chi-square test/Fisher’s exact test; ǂbased on Mann–Whitney U test.

27G, 27-gauge; 25G, 25-gauge; VP, vitrectomy probe.

### Intraoperative and postoperative Complications

The comparisons of complications between the two groups are shown in Table 3. There were no significant differences in iatrogenic retinal breaks or cataracts between the two groups. However, the 27G group had less hemorrhage and fewer applications of electrocoagulation (*P* = 0.004 and *P* = 0.022, respectively). During the 6-month follow-up after surgery, there were also no cases of infectious endophthalmitis or recurrent retinal detachment in any of the enrolled patients. Hemorrhage recurred throughout the entire follow-up period. Three eyes in the 27G group and two eyes in the 25G group underwent vitreous cavity lavage, and their postoperative visual acuity was significantly improved. The others were treated with medication, and it was found that the hematocele was gradually absorbed. Similarly, ocular hypertension (> 25 mmHg) and hypotension (< 6.5 mmHg) appeared in both groups at different time points. Most patients with an elevated IOP used eye drops to restore normal pressure. Two eyes in the 25G group were newly diagnosed with neovascular glaucoma and underwent successful antiglaucoma surgery. There was no significant difference in the incidence of all the above complications between the groups.

**Table 3:** Intraoperative and Postoperative Complications of the Two Groups

	27G beveled-tip (n = 25)	25G flat-tip (n = 22)	*p* value
Intraoperative complications			
Retinal break (number per operation) Iatrogenic hemorrhage (number per operation) Electrocoagulation (number per operation) Number of iatrogenic cataracts	0 (0, 1) 1 (0, 2) 0 (0, 0.5) 0 (0%)	1 (0, 2) 2.5 (1, 3.75) 1 (0, 2) 0 (0%)	0.238† 0.004ǂ 0.022ǂ
Postoperative complications			
Number of endophthalmitis Number of retinal detachments Number of vitreous hemorrhages 1 day after surgery 2 to 7 days 2 to 4 weeks 2 to 3 months 3 to 6 months Number of ocular hypotension 1 day after surgery 2 to 7 days 2 to 4 weeks 2 to 3 months 3 to 6 months Number of ocular hypertension 1 day after surgery 2 to 7 days 2 to 4 weeks 2 to 3 months 3 to 6 months	0 (0%) 0 (0%) 1 (4.0%) 2 (8.0%) 1 (4.0%) 2 (8.0%) 2 (8.0%) 4 (16.0%) 2 (8.0%) 0 (0%) 1 (4.0%) 0 (0%) 3 (12.0%) 5 (20.0%) 3 (12.0%) 2 (8.0%) 3 (12.0%)	0 (0%) 0 (0%) 1 (4.5%) 1 (4.5%) 0 (0%) 1 (4.5%) 4 (18.1%) 3 (13.6%) 0 (0%) 2 (9.1%) 0 (0%) 0 (0%) 3 (13.6%) 3 (13.6%) 2 (9.1%) 4 (18.1%) 2 (9.1%)	1.000† 0.609† 1.000† 0.609† 0.346† 0.856† 0.528† 0.414† 1.000† 1.000† 1.000† 0.536† 0.715† 0.346† 0.715†

†Based on chi-square test/Fisher’s exact test; ǂbased on Mann–Whitney U test.

27G, 27-gauge; 25G, 25-gauge.

### Changes in BCVA and IOP

**Fig. 3:**
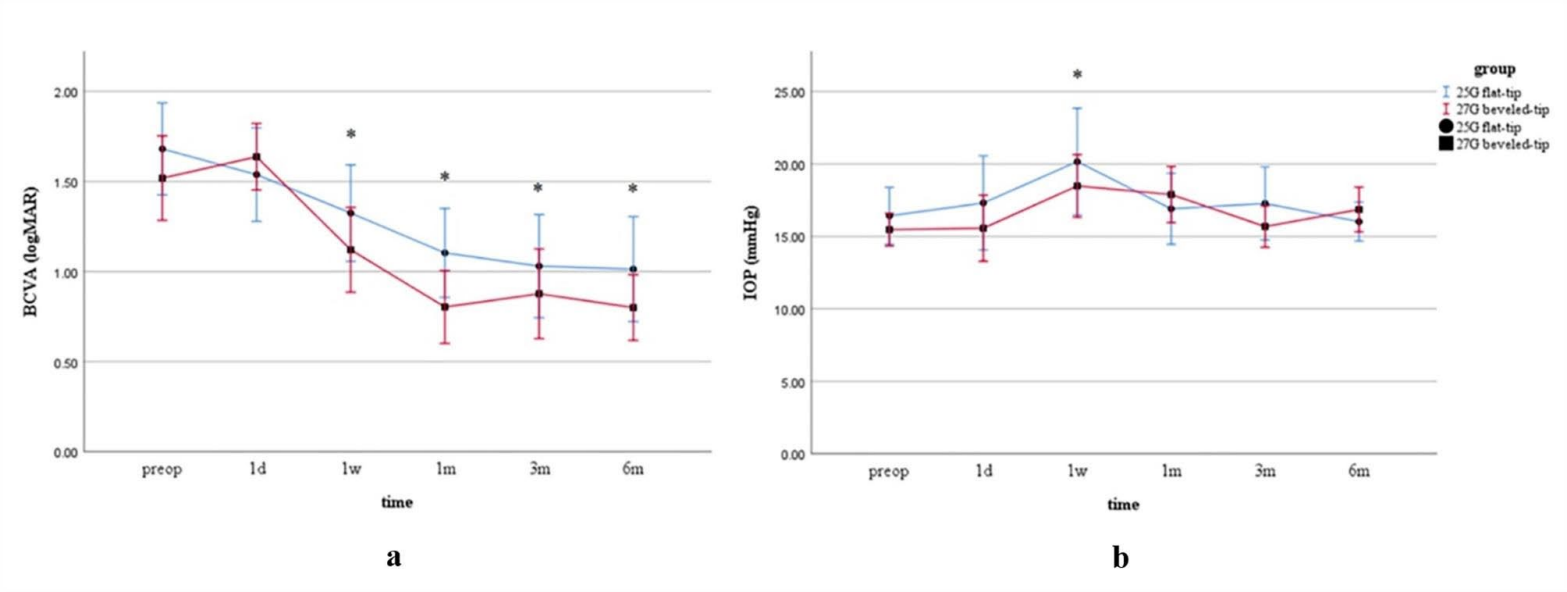
Time course of BCVA and IOP in the two groups. (**a**) is the change trend of mean BCVA at different time points from preoperative to postoperative 6 months. (**b**) is the change trend of mean IOP at different time points from preoperative to postoperative 6 months. * Indicates that the comparison between preoperative and postoperative measurements is statistically significant at *P* < 0.05

As shown in Table 4; Fig. 3, the postoperative BCVA of the patients in both groups was significantly improved at 1 week, 1 month, 3 months and 6 months compared with baseline (*P* = 0.001 and *P* < 0.001 in the last three periods). Nevertheless, the difference in BCVA improvement between the two groups was not statistically significant. The IOP in both groups was basically maintained at the normal level throughout, except for the first week after surgery when the IOP in both groups increased significantly (*P* = 0.017). However, it returned to normal on subsequent examination.

**Table 4:** Changes of BCVA and IOP in the Two Groups

	27G beveled-tip (n = 25)	25G flat-tip (n = 22)	*p* value (Preop vs. Postop)
BCVA (logMAR)			
Preop	1.53 ± 0.49	1.68 ± 0.66	
Postop (1d)	1.56 ± 0.42	1.54 ± 0.53	0.732
Postop (1w)	1.16 ± 0.62	1.32 ± 0.65	0.001
Postop (1 m)	0.82 ± 0.53	1.10 ± 0.47	< 0.001
Postop (3 m) Postop (6 m)	0.90 ± 0.60 0.80 ± 0.64	1.03 ± 0.58 1.01 ± 0.58	< 0.001 < 0.001
P value (27G vs. 25G)	0.584		
IOP (mmHg)			
Preop	15.5 ± 3.2	16.4 ± 5.5	
Postop (1d)	15.3 ± 5.3	17.3 ± 6.7	0.667
Postop (1w)	18.6 ± 6.1	19.6 ± 9.2	0.017
Postop (1 m)	17.9 ± 5.2	16.9 ± 5.2	0.078
Postop (3 m) Postop (6 m)	15.7 ± 3.8 16.8 ± 2.8	17.3 ± 2.7 16.0 ± 3.1	0.549 0.359
*P* value (27G vs. 25G)	0.412		

Comparisons between the two groups were based on GEE.

27G, 27-gauge; 25G, 25-gauge; BCVA, preoperative best-corrected visual acuity; logMAR, logarithm of the minimal angle of resolution; IOP, preoperative intraocular pressure; Preop, preoperative; Postop, postoperative.

## Discussion

Due to retinal neovascularization in PDR patients, vitreous hemorrhage and proliferative membranes occur frequently. The latter applies traction to the retina and easily results in retinal detachment [10]. At present, vitrectomy combined with proliferative membrane removal is the only effective approach to solve these serious problems [11]. Generally, if the attachment between the proliferative membrane and retina is loose, the VP tip can more conveniently reach under the membrane. Otherwise, the membrane must be separated from the retina by peeling the edge with auxiliary instruments at first [12]. At this time, improper techniques and actions may cause hemorrhage, iatrogenic retinal breaks or further expand the extent of retinal detachment [13].

Regarding MIVS for PDR treatment, most people believe that a smaller VP can more easily enter the gap below the proliferative membrane to achieve an accurate operation. However, some studies have shown that the traditional 27G vitrectomy system does not have many advantages over other systems [14–17]. The vitrectomy technique for PDR patients still needs further development. Recently, a new ultrahigh-speed vitrectomy system has been introduced. Its VP has a beveled design, and the port is closer to the tip. When the operator inserts the beveled tip between the layers and moves forwards, the membrane will be lifted up and aspirated into the port smoothly. This process was named the “shovel and cut technique” by its designer [7]. To date, only a few clinical trials on beveled-tip MIVS have been published [18, 19]. Hence, to evaluate advanced MIVS in the treatment of PDR, the results of this study are presented. In the 27G group, the productivity of cutting the membrane increased, and the number of VP exchanges to microforceps was obviously reduced. The surgeons also found that the use of the 27G beveled-tip VP avoided the need for the many repeated and ineffective surgical movements with the 25G flat-tip VP. Thus, we believe that the advanced VP could act as a multifunctional tool to improve the efficiency of membrane removal and avoid damage to the retina caused by frequent instrument replacement.

According to the existing clinical and fluidics studies, due to its small gauge, the conventional 27G vitrectomy system may take a longer time to cut the vitreous, which can also affect the overall surgical process [20–24]. The 27G beveled-tip vitrectomy system has the additional characteristics of an ultrahigh cutting rate, which can be up to 10,000 cpm. This improvement is intended to maximize the working efficiency of vitreous cutting. One article showed that the cutting efficiency of 27G systems might not be as high as that of 25G systems, but it has been greatly improved with the use of beveled-tip VPs over flat-tip VPs [8]. Our study also observed this finding. The difference resulting from the use of the 27G beveled-tip VP operating at 10,000 cpm and the 25G flat-tip VP operating at 7500 cpm in the removal of the core vitreous could be considered insignificant. However, we suspect that the vitrectomy time might still affect the total operation time, which was shorter in the 27G group but not significant.

In other aspects of the surgery, the proportion of eyes with postoperative scleral suture in the 27G group was significantly lower. This has also been reported in other studies on traditional MIVS systems [2, 25, 26]. Sutureless vitrectomy can optimize the surgical procedure, avoid patient discomfort caused by residual conjunctival sutures, and reduce corneal astigmatism after surgery. Therefore, the 27G beveled-tip MIVS is more advantageous in both surgery and patient recovery. In addition, there was no significant difference between the two groups in terms of the number of patients undergoing combined cataract surgery, the types of endotamponade used and intravitreous injection, which may indicate that the use of the advanced vitrectomy system would not cause more interference and affect the routine operation.

In terms of postoperative outcomes, compared with that at baseline, the BCVA of all patients gradually improved at 1 week, 1 month, 3 months and 6 months after surgery. The degree of improvement was similar between the two groups. Furthermore, except for a significant elevation at 1 week after the surgery, the IOP was maintained at normal levels at most time point. However, temporary intraocular hypertension is not a serious adverse event [27]. Overall, the use of 27G beveled-tip MIVS for treating PDR is not inferior to traditional 25G flat-tip MIVS. The two groups of patients achieved satisfactory treatment outcomes.

The safety of surgery is also a topic of concern for surgeons. According to the analysis of the data, the 27G group produced fewer hemorrhagic complications and needed less electrocoagulation, which could ensure a clear surgical field, as well as reduce retinal damage. During the 6-month follow-up period, there were no serious complications except for occasional recurrent vitreous hemorrhage. We believe that it is closely related to the patients’ blood glucose and other indicators, so more emphasis should be placed on patient education. To our surprise, the proportion of postoperative hypotension in the 27G group was slightly higher even though a smaller incision was made. We speculate that to ensure no serious leakage, more patients in the 25G group underwent scleral suturing. This may have been the reason for their IOP stability in the short term postoperatively. However, after observation with some potential intervention, the IOP of all patients returned to normal. The above results showed that the safety of the two vitrectomy systems was similar, and both of them can provide safe treatment for PDR patients.

Currently, there have not been many prospective comparative clinical studies on the treatment of PDR patients with beveled-tip MIVS. We found some unique advantages with this system in this specific operation and affirmed its effectiveness and safety. Moreover, we observed the productivity of cutting the membrane with a novel quantitative method. As described in previous literature, it was estimated based on the operation time, which might be affected by several confounding variables that could result in measurement error. This method can more objectively and visually present the results. However, there are also some limitations in this study. Due to the limited number of patients, we compared only two types of vitrectomy systems. Some ophthalmologists consider 25G MIVS as the first choice for surgery for PDR because of the faster vitreous cutting and greater hardness, so the 25G flat-tip MIVS was set as the control group. Therefore, the effect of gauge and cutting rate on the results cannot be excluded in the study. In addition, when we assessed the severity of the proliferative membrane at baseline, factors such as the degree of adhesion between the membrane and retina or the thickness of the membrane were not considered, which may have influenced the experimental outcomes.

## Conclusion

Compared with the 25G flat-tip MIVS, the 27G beveled-tip MIVS could be more efficient in removing the proliferative membrane while reducing the occurrence of intraoperative hemorrhages and electrocoagulation using appropriate surgical techniques and instrument parameters. Its vitreous removal performance was not inferior to that of the 25G MIVS and might offer potential advantages in total operation time. In terms of patient outcomes, the advanced MIVS demonstrates equal effectiveness and safety to 25G flat-tip MIVS.

## Data Availability

The datasets used and/or analysed during the current study are available from the corresponding author on reasonable request.
